# Role of aging in Blood–Brain Barrier dysfunction and susceptibility to SARS-CoV-2 infection: impacts on neurological symptoms of COVID-19

**DOI:** 10.1186/s12987-022-00357-5

**Published:** 2022-08-18

**Authors:** Daniel Adesse, Luis Gladulich, Liandra Alvarez-Rosa, Michele Siqueira, Anne Caroline Marcos, Marialice Heider, Caroline Soares Motta, Silvia Torices, Michal Toborek, Joice Stipursky

**Affiliations:** 1 Laboratório de Biologia Estrutural, Instituto Oswaldo Cruz, Fiocruz, Avenida Brasil, 4365, Pavilhão Carlos Chagas, sala 307b, Rio de Janeiro, RJ 21040-360 Brazil; 2 Department of Biochemistry and Molecular Biology, University of Miami Miller School of Medicine, Miami, FL 33136 USA; 3 Laboratório Compartilhado, Instituto de Ciências Biomédicas, Universidade Federal do Rio de Janeiro, Rio de Janeiro, Brazil; 4 Institute of Physiotherapy and Health Sciences, The Jerzy Kukuczka Academy of Physical Education, Katowice, Poland

## Abstract

COVID-19, which is caused by Severe Acute Respiratory Syndrome Corona Virus 2 (SARS-CoV-2), has resulted in devastating morbidity and mortality worldwide due to lethal pneumonia and respiratory distress. In addition, the central nervous system (CNS) is well documented to be a target of SARS-CoV-2, and studies detected SARS-CoV-2 in the brain and the cerebrospinal fluid of COVID-19 patients. The blood–brain barrier (BBB) was suggested to be the major route of SARS-CoV-2 infection of the brain. Functionally, the BBB is created by an interactome between endothelial cells, pericytes, astrocytes, microglia, and neurons, which form the neurovascular units (NVU). However, at present, the interactions of SARS-CoV-2 with the NVU and the outcomes of this process are largely unknown. Moreover, age was described as one of the most prominent risk factors for hospitalization and deaths, along with other comorbidities such as diabetes and co-infections. This review will discuss the impact of SARS-CoV-2 on the NVU, the expression profile of SARS-CoV-2 receptors in the different cell types of the CNS and the possible role of aging in the neurological outcomes of COVID-19. A special emphasis will be placed on mitochondrial functions because dysfunctional mitochondria are also a strong inducer of inflammatory reactions and the “cytokine storm” associated with SARS-CoV-2 infection. Finally, we will discuss possible drug therapies to treat neural endothelial function in aged patients, and, thus, alleviate the neurological symptoms associated with COVID-19.

## Introduction

At the end of 2019, the world faced the beginning of what would become the biggest pandemic in recent history. In the province of Wuhan in China, the first cases of a new severe acute respiratory syndrome (SARS) were reported. The etiological agent of such syndrome was shown to be a coronavirus, named SARS-CoV-2 and the disease was then referred to as coronavirus disease-19 (COVID-19). In less than 2 years, it has already caused more than 4 million deaths worldwide. Patients affected by COVID-19 may be asymptomatic or have mild symptoms, with fever, loss of smell, taste, body pain. Disease can progress to a severe form where the need for hospitalization occurs, which main symptom is a respiratory distress due to pulmonary insufficiency. Age has also been shown to be a critical factor for COVID-19 aggravation and hospitalization. Among the symptoms observed in hospitalized patients, impaired neural functions have been reported, such as cases of acute cerebrovascular disease with episodes of stroke, headaches, dizziness, loss of consciousness, ataxia, and epilepsy.

SARS-CoV-2 is capable of infecting human cells through the binding of its surface protein Spike1 to host cell surface proteins. The angiotensin converting enzyme-2 (ACE2) functions as a receptor for the virus and its main gateway. Pulmonary, cardiac, gut cells and body vasculature express high levels of ACE2. In the vascular endothelia, ACE2 plays essential roles in the Renin–Angiotensin system, regulating arterial pressure by mediating the conversion of angiotensin II (a vasoconstrictor) to angiotensin (1–7) (a vasodilator). Another receptor, the transmembrane protease serine type 2 (TMPRSS2) has been shown to cleave Spike protein and mediate membrane fusion by promoting the internalization of the virus in cells, thus acting as a co-receptor facilitating the process of viral infection. Several data have attributed the worsening of the condition in patients with the induction of vascular dysfunction in the body, where processes such as coagulation and blood pressure control fail, vascular inflammation takes place, and the cytokine storm promoted by the immune response contributes to severe pathology, leading many individuals to death. These events have been reported to be more intense in elderly patients and critical to disease progression.

In the brain, several routes for viral entry have been proposed, including the olfactory nerve, the choroid plexus and the blood–brain barrier (BBB). The role of the BBB appears to be of utmost relevance, given that SARS-CoV-2 can be found free in the bloodstream [1] suggesting that viral particles could reach BBB cells. In the brain, endothelial cells of blood capillaries contact other cells of the neurovascular unit, especially astrocytes and pericytes, forming the basic unit of the BBB [2, 3]. A significant number of COVID-19 patients, regardless of the severity of the respiratory disease, have shown to present impaired neurological functions [4, 5], being more disruptive and lethal in elder individuals [6]. Biomarkers of the Central Nervous System (CNS) damage such as neuronal neurofilament and astrocytic GFAP proteins were found to be increased in serum and cerebrospinal fluids of patients with COVID-19-induced neurological syndrome [7]. SARS-CoV-2 particles has been detected in the cerebral cortex of patients [8, 9] and specifically in astrocytes [10]. COVID-19 also leads to a thinning of the cortical tissue, which correlates with the neuropsychiatric symptoms [10]. Moreover, a recent study in UK described that COVID-19 patients displayed a reduced global brain size, with a marked reduction in grey matter thickness in the orbitofrontal cortex and parahippocampal gyrus, and markers of tissue damage that correlated to the primary olfactory cortex [11].

Aging is characterized by a decline of biological functions, leading to cellular senescence. In the CNS, endothelial and astrocytic senescence have been demonstrated to lead the BBB to a dysfunctional state in neurodegenerative diseases [12]. Still, little is known about the effects of SARS-CoV-2 on the function and structure of the aged BBB, such as the expression levels and organization of tight junction and transport proteins such as P-glycoprotein, MRP, RAGE, LRP and the profile of cytokine secretion after endothelial activation. Even less is known whether BBB dysfunction induced by viral infection can be propagated to the neural tissue and thus be the triggering mechanism of the neural dysfunctions that promote the establishment of the neurological symptoms reported in patients with COVID-19.

In this review, we will discuss (1) the interaction of SARS-CoV-2 with the BBB endothelium and the impact of these interactions on neurovascular functions; (2) the profiles of expression of potential SARS-CoV-2 receptors in human BBB cells, with a focus on ACE2, comparing the expression patterns in the context of aging; (3) the immune response in aging and its role of SARS-CoV-2 on BBB dysfunction and neuroinflammation; and (4) the role of endothelial dysfunction as a trigger to neurological symptoms. Finally, we will discuss (5) possible drug therapies to treat neural endothelial function in aged patients, and, thus, alleviate the neurological symptoms associated with COVID-19.

## Effects of SARS-CoV-2 on neural endothelium

Several reports have dedicated attention to identify potential routes of SARS-CoV-2 transmission into the CNS. They have been proposed to involve the nasal and oral mucosa, enteric epithelial wall, and the BBB [13, 14].

The capillaries that vascularize the CNS exhibit a series of physiological properties that tightly control the transport of cells, nutrients and metabolites between the blood and the brain parenchyma (reviewed in [3]). Such characteristics make this structure act as a protective barrier widely known as BBB. Cerebral capillaries are composed by microvascular endothelial cells (BMECs), which are organized in a juxtaposed way, connected via tight junction proteins, and surrounded by cellular components: (i) pericytes—mural cells enwrapping capillary blood vessels on their abluminal side and (ii) astrocytes, whose endfeet cover most of the vasculature surface area; and non-cellular components, including (iii) the basement membrane (BM), which provides structural support for the cellular components and functions as an intercellular communication hub (reviewed in [15]). All these elements serve as the interface between endothelial cells, microglia, and neurons that together originate the Neurovascular Unit (NVU) of the BBB, whose main functions in the CNS are regulation of homeostasis and protection from blood-borne toxins, pathogens, inflammation, and injury [16].

The endothelium activity is regulated through a wide repertoire of receptors present in its membrane, as well as through specific proteins and junction receptors that govern intercellular and endothelial-matrix interactions [17]. The cerebral endothelial cells (ECs) are even more specialized to restrict the paracellular and transcellular movement of solutes. Their junctions are composed of a variety of specialized junctional regions known as *adherens* and tight junctions (TJs) that are built by* adherens* and tight junction proteins, ensuring the integrity of the vascular tube and regulation of the traction forces that are important for BBB integrity [18]. CNS TJs are specialized in their molecular and structural composition, and the specific combination of TJ proteins at the BBB determines its paracellular permeability (reviewed in [3]). Brain ECs form a continuous lining that lacks fenestrations and have low levels of transcytosis, properties that greatly limit transcellular permeability [19]. The study of the biology of microvascular ECs is particularly important and represents the central structure for understanding the molecular mechanisms of invasion and viral infection in the CNS.

The BBB has been a target of discussion as a critical invasion route for SARS-CoV-2 into the brain parenchyma. A circulating virus or pathogen can invade the brain parenchyma through the damaged BBB and leaks into the interstitial fluid, and then enters the cerebral spinal fluid through the glymphatic system. Viruses in the blood can also enter the fourth ventricle directly through a damaged blood–CSF barrier. Indeed, microglia and astrocytes were found to be infected by murine coronavirus (MHV-A 59, [20]), which provide further evidence of a crosstalk between the glymphatic system and COVID-19 [21]. However, BBB-mediated viral entry into the CNS may not necessarily depend on capillary endothelial wall disruption, since different mechanisms of pathogen transport across endothelial lamina of the capillaries have also been described [22].

SARS-CoV-2 has been identified in brain capillary endothelium obtained from a post-mortem COVID-19 case [23]. The interaction between Spike proteins with endothelial cells by using in vitro BBB models showed significant changes to barrier properties. It was demonstrated that SARS-CoV-2 Spike protein induced destabilization of BBB-related tight junctions and promoted pro-inflammatory status in human BMECs [24]. Another SARS-CoV-2 protein, its main protease (M^pro^), led to clevage of NEMO, a member of the NF-κB pathway, further contributing to an inflamed BBB state. Additionally, an increasing number of case reports have emerged describing acute neurological disorders as an implication of SARS-CoV-2 infection, suggesting that SARS-CoV-2 crosses the BBB (Ng Kee Kwong et al. [25]). We have recently described that BBB cells display a unique profile of SARS-CoV-2 receptors, with differential expression of molecules such as ACE2, TMPRSS2, ADAM17 and others in human BMECs, astrocytes, pericytes, microglia and neurons [26]. We have also demonstrated that SARS-CoV-2 leads to minimal productive infection on immortalized HMECs [27], although increased apoptosis and inflammation was triggered in infected cultures. Experimental infection of K18-hACE2 mice or hamsters led to effective infection of BMECs, despite no changes in tight junctions were observed [28].

In non-pathological conditions, ECs control blood flow, in part, by maintaining an antithrombotic microenvironment on its luminal surface, which facilitates the transit of blood components. Disorders resulting from inflammatory processes alter this activity and tissue homeostasis, generating a microenvironment with a thrombotic profile (reviewed in [29]). Several risk factors for cardiovascular diseases such as diabetes, hypertension, smoking and obesity can cause changes in blood flow and consequently generate endothelial dysfunction, resulting in a thrombotic profile, along with increased permeability and secretion of pro-inflammatory cytokines, apoptosis, de-differentiation, and metabolic changes (reviewed in [30]).

Recent evidence points to important vascular changes during SARS-CoV-2 infection. The vascular symptoms of COVID-19 share pathophysiological mechanisms and phenotypes compatible with endothelial dysfunctions, being the most common disorders of coagulation, thrombosis and inflammation of multiple organs resulting from changes in vascular permeability ([31, 32], reviewed in [33]). Infection alone has the potential to cause significant endothelial damage. In contrast, patients with pre-existing cardiovascular diseases who already have endothelial dysfunctions are more susceptible to worsening due to SARS-CoV-2 infection [34]. Microvascular infarcts and hemorrhages, due to SARS-CoV-2 infection, are probably also critical in the development of encephalopathy, and other neurological manifestations of COVID-19 [35]. In clinical studies to evaluate pro-thrombotic markers during SARS-CoV-2 infection, it was demonstrated that circulating platelets have a higher expression of specific activation markers, such as P-selectin (CD62P), LAMP-3, and GPIIb/IIIa in patients with COVID-19 compared to healthy donors. Moreover, platelets exhibited hyperresponsive behavior with increased aggregation and adhesion response, which might be linked to increased expression of adhesive receptors, such as von Willebrand factor (VWF) and fibrinogen receptors, GPIbα/GPIX and GPIIb/III, identified in patients with COVID-19 [36–39]. Infected patients also present elevated levels of d-dimer, a blood clotting marker that has been considered an indicator of prognosis in SARS-CoV-2 infection (reviewed in [40]). Like d-dimer, a marked elevation of factor V activity was observed in severe COVID‐19, and it was associated with venous thromboembolism [41].

The “cytokine storm syndrome” (CSS) has been identified as a central event in COVID-19 and is presumably the main cause of the observed endothelial damage and the establishment of acute respiratory distress syndrome (ARDS). This clinical condition is characterized by an excessive release of proinflammatory cytokines including tumor necrosis factor-α (TNF-α), granulocyte–macrophage colony-stimulating factor (GM-CSF), monocyte chemoattractant protein 1 (MCP1), interleukin-1α (IL-1α), IL-1β and IL-6. In more severe cases, increased levels of IL-2, IL-7, IL-10, and TNF-α were observed, which may indicate an important role of specific cytokines and chemokines in driving COVID-19 progression [42–44]. This unrestrained increase in the levels of these cytokines ultimately results in the influx of immune cells from the circulation to the infection sites. The secreted chemokines activate neutrophils that secrete high levels of peroxidase and reactive oxygen species, and activate matrix metalloproteinases (MMPs), which aggravate damage to lung tissue and the cardiovascular system. Such immune hyper-activation can be particularly destructive to the tissues as it generates a destabilization of the interactions between endothelial cells, with damage and increased permeability of the vascular barrier that can trigger the failure of multiple organs (reviewed in [45]). Zuo et al. [46] showed that neutrophil extracellular traps (NETs) are enhanced in hospitalized COVID-19 patients. The same authors also documented that sera from individuals with COVID-19 trigger NET release from control neutrophils in vitro. NETs are extracellular webs released by neutrophils in response to infections. These traps are basically made up of DNA, histones, microbicidal proteins and oxidizing enzymes. However, this is a suicidal response, because when not properly regulated, NETs can attach to the capillary endothelium and platelets, induce coagulation and thus initiate the spread of inflammation and thrombosis with alveolar-capillary barrier damage, leading to vascular leakage, edema and finally ARDS [46–50].

Another factor that deserves attention is related to the establishment of macrophage activation syndrome, one of the mechanisms strongly identified as responsible for endothelitis in patients with the severe COVID-19 form ([14, 51, 52], reviewed in [53]). In a comparative study of lung tissues of patients who died from COVID-19 or ARDS due to H1N1 infection, a greater number of ACE2-positive ECs and significant changes in endothelial morphology were observed [54], drawing attention to the presence of intercellular junction’s rupture, edema and loss of contact with the basement membrane.

Vascular alterations, such as endothelial rupture by direct signaling effects or indirectly by increasing the production of inflammatory mediators accompanied by coagulation cascade dysregulation, have been reported in infections with other coronaviruses [55, 56]. In fact, the coagulation phenotype of COVID-19 is so remarkable that it was proposed that this disease should be in fact named as viral thrombotic fever [57]. Similar to COVID-19, SARS-CoV infection was associated with endothelial dysfunction, thrombotic complications, and hematological manifestations. As observed in SARS-CoV-2 infection, SARS-CoV also hijacks ACE2 as the main receptor for entry into host cells and, therefore, it specially targeted pneumocytes and enterocytes due to the high expression of ACE2 by these cells [58, 59]. Cases of vasculitis and EC inflammation have been documented, as well as pulmonary embolism, deep vein thrombosis and generalized multiple organ infarctions associated with polyangiitis and microcirculatory disorders in post-mortem patients with SARS-CoV infection. Additionally, SARS-CoV was also associated with fetal complications due to dysfunction of the placental circulation [60–62]. Likewise, MERS-CoV infection also altered coagulation pathways. Patients infected with MERS-CoV presented thrombocytopenia, a drop in platelet count and, in the most severe cases, intracerebral hemorrhages and multiple organ failure. [63–65]. Experimentally, the effect of MERS-CoV on the coagulation cascade has been observed in transgenic mice expressing human dipeptidyl peptidase 4 (hDPP4), which has been identified as a target receptor for virus entry and binding to the host cell. In these animals, histopathological analyzes revealed the presence of microthrombi in the lung vasculature, as well as inflammatory infiltrates and alveolar edema [66].

## The role of aging on BBB function and expression of SARS-CoV-2 receptors

Clinical observations indicate that elderly COVID-19 patients are the most affected individuals by the disease progression and that SARS-CoV-2-infected patients aged over 80-years old showed a greater risk of death in comparison with younger patients [67, 68]. Initial reports described death rates above 10% in people older than 70 [69, 70]. Several key receptors that regulate SARS-CoV-2 entry into the host cells are observed in older patients [71]. In addition, elderly people have a less efficient response to vaccinations and worse outcomes from cancer or infectious diseases, likely due to immunosenescence [72]. For example, older individuals show diminished type-1 IFN production upon vaccine administration [73], which is a key cytokine in immune response to multiple viruses including SARS-CoV-2 [74]. On the other hand, it is also possible that age by itself may not necessarily be a risk factor for severe COVID-19 outcome, but rather that diseases more commonly found in elderly patients are aggravating factors themselves.

In an attempt to find pharmacological targets of interest for COVID treatment, studies of the molecular mechanisms by which SARS-CoV-2 can infect human cells have been extensively pursued. As mentioned above, ACE2 is responsible for degrading angiotensin II and blood pressure regulation [75]. ACE2 is highly expressed in different organs such as kidney, heart, lungs, small intestine, liver and including brain [76–80]. In the CNS, ACE2 can be found in neurons, astrocytes and oligodendrocytes, with high expressions in the cortex, substantia nigra and in paraventricular areas, such as the choroid plexus of the lateral ventricle [68, 81]. Endothelial cells from blood vessels in resting (“healthy”) conditions express lower levels of this receptor, and are potentially less susceptible to SARS-CoV-2 infection when compared to other neural cell types [82, 83]. On the other hand, the invasion of human cells by SARS-CoV-2, which occurs mainly through endocytosis, does not rely solely by its interaction with ACE2, but rather involves interactions of viral proteins with other host cell proteases [84]. Thus, the expression levels of ACE2 do not fully determine the susceptibility to SARS-CoV-2 infection because the invasion also depends on the availability of proteases in the host cell that are responsible for the protein S cleavage, allowing the viral envelope fusion with the target cell [85].

One of the proteases that allow SARS-CoV-2 cell invasion is TMPRSS2, which is a member of the hepsin subfamily of membrane anchored serin proteases. It has been suggested that TMPRSS2 cleaves SARS-CoV-2 Spike1 protein, therefore allowing viral entry into human cells. TMPRSS2 is expressed in several tissues including the lungs, heart, kidney, liver, colon, esophagus and brain [68]. In the CNS, TMPRSS2 appears to be mostly co-expressed with ACE2 in glial cells [82]. In a similar fashion to ACE2, TMPRSS2 expression is lower in children and increases with age [71]. Accordingly, ACE2 expression in the upper and lower airways is significantly lower in children when compared to adults [71]. Age was a significant factor for ACE2 and TMPRSS2 expression in alveolar, bronchiolar, renal and hepatic tissues in elder mice [86] and in alveolar type II cells in elder humans [87]. Moreover, stromal immune-inflammatory cells in humans over 50 years old display increased ACE2 and TMPRSS2 expression [88]. Besides age-related expression levels of ACE2, males appear to expresses this receptor to a higher degree than females [89]. In this context, epidemiological data also suggest that older males may be at a higher risk group for severe COVID-19 [90].

A disintegrin and metalloproteinase domain 17 (ADAM17) is another potential target of interest in COVID-19 research. ADAM17 has been identified in lungs, skeletal muscle, heart, ovaries, testis, pancreas, kidneys, small intestines and brain [91, 92]. In the CNS, ADAM17 is mostly found in astrocytes and endothelial cells from the BBB [26, 93]. ADAMTs are part of the metzincin metalloprotease family, which also includes matrix metalloproteases and snake venom metalloproteases [94]. Its main function in healthy individuals is to cleave extracellular proteins altering their activity, such as membrane bound TNF-α into its soluble form [95]. ADAM17 also cleaves APP, suggesting a role in Alzheimer’s disease pathogenesis [96]. Additionally, ADAM17 was found to be selectively increased in CSF of patients with neoplastic meningitis [97]. The mechanism by which ADAM17 promotes SARS-CoV-2 infection is yet to be elucidated, however, it is possible that its protease activity can promote the viral particle fusion with the cytoplasmic membrane [98]. Moreover, the role of ADAM17 may be also related to its ability to promote IL-6 and other inflammatory pathways [99], since IL-6 receptor is a known substrate for ADAM17 [94], a mechanism that will be further discussed in “Aging and mitochondrial dysfunction in BBB neuroinflammation” section of this review. ADAM17 is increased in aging in mice and contributes to vascular remodeling and impaired endothelial wall shear stress mechanosensing [100]. Interestingly, in *Drosophila*, ADAM17 showed a protective role in neuronal and glial degeneration in the aging retina [101].

Additional proteins that may be involved with SARS-CoV-2 infection are cathepsins, CD209L and furin. Cysteine protease cathepsins (Cat) function in protein degradation in lysosomal systems, but can also participate in the plasma membrane signaling and in extracellular matrix proteins regulation, therefore impacting functions such as endocytosis [102]. This lytic activity and promotion of endocytosis has been observed with SARS-CoV [103] and some of them, such as Cat-L and Cat-C, have shown promising initial results as targets in initial pharmaceutical trials [8, 9, 104]. Interestingly, cathepsins are closely related to the TGF-β signaling pathway [105], which plays a crucial role in the maintenance of the BBB integrity.

CD209L is a member of the calcium dependent family of lectins, whose main function is to mediate protein interactions with mostly carbohydrate domains, but also with other proteins, lipids and nucleic acids [106]. While the interaction of CD209 with SARS-CoV-2 has yet to be thoroughly studied, its interaction with other viruses has been show before, including SARS-CoV. CD209L works synergistically with ACE2 promoting internalization of viral particles [107–109].

Furin is a protease, which participates in cleavage of proteins and alterations of their substrates’ functions. Furin has been shown to participate in the activation of several factors such as hormones, neuropeptides and other signaling molecules [110]. In the context of COVID-19, some targets of interest that are activated by furin are IFN-γ and ADAM17 [111]. In a similar fashion, furin may also work in conjunction with ACE2, TMPRSS2 and other proteases facilitating viral entry into host cells [112].

## Immune response in the aged brain: SARS-CoV-2-induced neuroinflammation

The knowledge about the neural sequelae in COVID-19 is still relatively sparse and new insights and studies are highly needed. The mechanisms underlying the SARS-CoV-2-induced neurological symptoms of COVID-19 and the impact of aging are subjects of intense investigation. In general, examples of the contribution of hallmarks of aging to the age-related predisposition to COVID-19 include:

- Age-related mitochondrial dysfunction, that can induce epigenetic changes in regulatory T-cells (Treg), which impair their pro-recovery functions to hinder proper resolution of inflammation and repair.
- Monocytes and naïve T-lymphocytes undergo cellular senescence following telomere attrition from sustained replication, impairing the host’s ability to mount an efficient immune response to a viral challenge or create a memory T-cell response to vaccines.
- Altered intercellular communication underlies the low-grade inflammation associated with aging, which contributes to the development of age-related comorbidities (reviewed by [113])

The ability to control viral load is one of the best prognostics of whether a patient will have mild or severe COVID-19 symptoms [114]). For the immune system to effectively suppress and eliminate SARS-CoV-2, it must perform four main tasks: recognize, alert, destroy and clear the virus. Each of these mechanisms is known to be dysfunctional and increasingly heterogeneous in older people [115]. However, which of these tasks is the most relevant to COVID-19 progression in older people is not yet clear ([116], reviewed by [117]).

During aging, the immune system changes in two major ways. One is a gradual decline in immune function called *immunosenescence*, which hampers pathogen recognition, alert signaling, and clearance. This mechanism is not to be confused with cellular senescence, an aging-related phenomenon whereby old or dysfunctional cells arrest their cell cycle and can become epigenetically locked into a pro-inflammatory state in which they secrete cytokines and chemokines. A chronic increase in systemic inflammation called *inflammaging* is an important hallmark of aging. Inflammaging is characterized by a chronic sterile low-grade inflammation, which is an overactive and less effective alert system [118]. A variety of cellular and molecular mechanisms are involved in inflammaging, which include cellular senescence, immunosenescence, mitochondrial dysfunction, meta-inflammation and gut dysbiosis (reviewed by [14]). Senescent cells have a differential secretory profile that includes increased release of pro-inflammatory mediators such as IL-6, IL-8, IFN-γ, MCP-1, and ECM-degrading molecules, including MMP2 and TIMP2, as well as increased expression of cell cycle regulators [119].

A growing body of evidence suggests that COVID-19 overall severity in older patients may be related to immunosenescence and inflammaging. In the case of neurological manifestations of COVID-19 such factors may also play a pivotal role in neuropathology. Inflammaging is a known risk factor for dementia, stroke, and cerebral small vessel disease (CSVD) [120]. Aging is also known to potentiate brain pathology in viral infections, such as West Nile Virus infection, in which patients with > 60 years old have a 20-fold increase in the risk of developing neurological manifestations and retinopathy [121, 122].

Inflammaging, as a phenomenon of sustained systemic inflammation, contributes to increased BBB permeability [123], which may account for higher rates of SARS-CoV-2 invasion of the brain parenchyma. Although endothelial cells express lower levels of ACE2 receptors and low susceptibility to SARS-CoV-2 infection [124], it has been shown that upon inflammatory stimulus, ACE2 expression can be upregulated, thus increasing infectivity [125]. In fact, apart from the well-known cytokine storm, COVID-19 patients with inflammatory neurological disease or encephalopathy, had specific increase in circulating IL-6, IL-8 and TGF-β1 [126]. Therefore, inflammaging could not only disrupt BBB integrity but also increase endothelial cell susceptibility to SARS-CoV-2 infection.

The host immune response of COVID-19 presents a signature called “the global immune signature” of SARS-CoV-2 infection, which consists of elevated serum cytokines (particularly IL-1β, IL-6 and TNF-α), impaired IFN responses, and peripheral lymphopenia as markers of severe disease [127, 128].

The aging brain is also vulnerable to inflammation, as demonstrated by the high prevalence of age-associated cognitive decline and Alzheimer’s disease [129–131]. As mentioned above, aging is characterized by the development of persistent pro-inflammatory responses that contribute to atherosclerosis, metabolic syndrome, cancer and frailty [132–134]. Systemically, circulating pro-inflammatory factors can promote cognitive decline [135, 136]. The underlying mechanisms that initiate and sustain maladaptive inflammation with aging are not well defined (reviewed by [137]); however, they appear to include microglia losing their ability to clear misfolded proteins that are associated with neurodegeneration [138, 139].

As stated above, epidemiological data suggest that older males may be at a higher risk for severe COVID-19. This observation correlates with the fact that sex may be indeed a predictor for some neuropathological events. Sex-differences are now widely described as an important factor in aging in the context of cerebral microvasculature physiology and response to insults. Sex hormones androgens [testosterone (T) and dihydrotestosterone (DHT)], estrogens [estradiol (E2), estrone, estriol], and progestins [(e.g., progesterone (P4)] are produced primarily in the gonads but can also be produced by vasculature and in the brain, where they may act as important regulatory factors [140]. As such, sex hormones can mediate gap junction communication, vascular relaxation, and neuronal plasticity (reviewed by [141]). However, hormonal production decline observed mostly after menopause correlates with aging and cerebrovascular dysfunction. Both estrogen and androgen receptors were found to be expressed in brain microvascular endothelial cells, in males and females [142]. As recently reviewed by Robison and colleagues [141], cerebral blood flow is higher in girls (4–8 years old) than boys and this effect is still present in elder females. Interestingly, hormone replacement therapy in post-menopausal women restored the decrease found in whole cerebral and cerebellar blood flow [143].

Considering age and sex as risk factors for neuropathologic conditions, being Neuro-COVID-19 included, is corroborated by vast literature that reports increased cytokine transport through the BBB [144, 145], astrocyte reactivity [146] and Pgp activity [147] as function of sex and age differences. Also, females have better BBB integrity in humans [148] and respond differently to ApoE4 and high fat diet in mice [149]. Adult females also respond to stroke with smaller infarct areas than males in animal models, and such effect was abolished when females were ovariectomized (reviewed by [146]).

## Aging and mitochondrial dysfunction in BBB neuroinflammation

It has been extensively proven that a large array of neurological diseases and brain aging itself are associated with oxidative stress [150–152]. Multiple sclerosis, stroke, brain tumors, and neuroinfections are conditions which associate both reactive oxygen species (ROS) aggression and BBB impairment as well-proven pathogenic mechanisms [137]. Whether oxidative damage is an important and early event in BBB alteration process, has not been fully established so far. However, BBB disruption was reported not only in vascular or inflammatory brain diseases but also in neurodegenerative disorders in which oxidative stress plays an important role in the pathogenic scenario [153, 154].

Mitochondrial dysfunction has been implicated in the establishment and/or progression of neurological and neurodegenerative diseases [155]. In the aging process, mitochondria accumulate replication errors in their DNA (mtDNA) and oxidative damage from the production of reactive oxygen species (ROS) (Park and Larsson [156]). In addition, toxic exposure and pathological processes, including diabetes, cardiovascular diseases, gastrointestinal disorders, and cancer can also lead to mitochondrial dysfunction [157–159]. A recent study demonstrated that SARS-CoV-2 infection of lung cells induced mitochondrial disruption, which was correlated with poor immune response and exacerbated inflammation leading to COVID-19-related sepsis [160].

SARS-CoV-2 infection impacts cell stress responses and redox balance (reviewed by [161]). It has been described that SARS-CoV-2 leads to disruption of redox balance in infected cells through modulation of NAD+ biosynthesis, PARP function along with altering proteasome and mitochondrial functions. ROS production and the increase in IL-6 production and lipid peroxidation that ensues, contribute to cytotoxicity. These events are related as enhanced ROS production hampers the proteasome function, which then leads to impaired protein degradation and further negatively influence mitochondrial function [162–165].

Disturbances in the permeability of the BBB is a common factor in several neurological disorders that can impact the oxidative balance. Oxidative stress influences pathophysiological processes such as cardiovascular diseases, neurodegenerative diseases, chronic neuroinflammation, Alzheimer’s disease, and even aging [166]. Mitochondria are among the major cellular sources of ROS through the activity of the electron transport chain [167]. Declines in glial cell functions have been described during aging, which was accompanied by mitochondrial dysfunction and inflammation. Inflammatory mediators released by activated glial cells can modulate mitochondrial function, thereby establishing a crosstalk between mitochondrial dysfunction and neuroinflammation [168]. Mitochondrial DAMPs may have the dual role of mediating neurodegeneration and amplifying neuroinflammation. These events are further impacted by environmental risk factors and can further contribute to an increase in ROS production [169, 170]. ROS accumulation by mitochondria is a major cause of neuronal apoptosis and impaired ROS clearance can alter mitochondrial viability and alter BBB permeability [171]. Indeed, chronic oxidative stress can disrupt electron transport chain activity [172], that could be propagated by microglia and astrocytes, being the main inductors of hyperinflammation and neuronal loss [173]. Increased ROS levels induces transcription of pro-inflammatory genes and release of IL-1, IL-6, IL-10 and TNF-α which, together, generate a favorable microenvironment to neuroinflammatory processes [170].

Given the importance of mitochondria in the antiviral defense and their participation in the immune system, mitochondrial dysfunction involving alterations of the mitochondrial respiratory chain, production of mtROS and regulation of cell death may be potential targets in the pathogenesis of COVID-19 [174–176]. In addition, mitochondrial dysfunctions leading to the loss of BBB integrity was suggested to be involved in SARS-CoV-2 trafficking into the brain [177]. Because BBB functions decline during aging [178], elderly population has an inflammatory BBB, thus providing a favorable microenvironment for neurological manifestations arising from the “cytokine storm”, which may also be a direct consequence of mitochondrial dysfunction. Therefore, understanding the effects of neuroinvasion in the elderly population is of great importance to elucidate the mechanisms by which viruses cause this disorder in the CNS and also to design an effective treatment that improves the clinical outcome of COVID-19. Antioxidant system declines with aging and this decline correlates with decrease in mitochondrial electron transfer, which, in turn, favors generation of ROS [179, 180]. Endogenous antioxidants such as coenzyme Q10, glutathione and melatonin are, therefore, potential and promising targets for anti-aging therapy and could prevent brain inflammaging effects [181]. In that direction, we have shown that a targeted delivery of COQ_10_ prevented cellular senescence and oxidative stress in neural progenitor cells and astrocytes treated with antiretroviral drugs, exposed to drugs of abuse or infected with HIV-1 [182, 183]. Moreover, exogenous melatonin treatment prevented neuroinvasion and cerebrovascular abnormalities in K18-hACE2 mice [184], thus corroborating a protective role of antioxidant therapy in Neuro-COVID-19.

Cytokines produced during the inflammatory course of COVID-19 induce mitochondrial dysfunctions such as increased permeability, prevent mitochondrial oxidative phosphorylation, enhance ROS production, and induce alterations of mitochondrial dynamics and even apoptosis [185–187]. Moreover, pathological impact of SARS-CoV-2 involves targeting mitochondria and mitochondrial failure [188–190]. Mechanistically, SARS-CoV-2 infection has been found to impact host mitochondrial functions through ACE2 regulation and open-reading frames that allow for increased viral replication and evasion of host cell immunity [191]. Because reprogramming of mitochondria is a strong inducer of oxidative stress and inflammatory reactions, targeting ROS production as adjuvant therapy with anti-oxidants could decrease excessive inflammation and cell damage that lead to severe SARS-Cov-2 infection.

Mitochondria are dynamic organelles that control its metabolism and interaction with other organelles via two major events that occur simultaneously in the organism, namely mitochondrial fission and fusion. Proteins that control mitochondrial fission include Drp1 and Fis1 [192, 193] and mitochondrial fusion includes Mitofusin 1 and Mitofusin 2 (Mfn1 and Mfn2) and OPA1. Mitochondrial morphology is highly dynamic, being different and adjustable in each cell type and maintains a genetic and biochemical homogeneity by allowing for dilution of toxic superoxide species, mutant mtDNA, and repolarization of membranes during homeostasis, stress and inflammation [194–197]. Accordingly, we have recently shown that infection of HBMEC by SARS-CoV-2 leads to an inflammatory status with cytokine and chemokine production and remodeling of mitochondrial networks, with increased Mfn2 expression [27]. However, additional studies that focus on the role of host cell mitochondrial remodeling upon SARS-CoV-2 infection, including mitochondrial biogenesis and metabolic responses, remain to be elucidated.

## Endothelial dysfunction as a trigger to neuroinflammation and neurological symptoms

Loss or alterations in the perception of taste and smell were the first unconventional symptoms described in COVID-19 patients. They appear to be prevalent in at least 19% of patients, although they could possibly be present in as much as 70% of infected patients [198–200]. These symptoms are more common in the early stages of the disease and do not seem to last long in non-severe patients [199–201]. Other common and possibly related symptoms of COVID-19 are nausea, headaches, short-term memory disruption, lack of attention, disorientation and irritability [202–204]. Despite being debilitating, they usually are not life-threatening. On the other hand, there have been reports of far more dangerous outcomes of SARS-CoV-2 invasion into the CNS, including encephalitis. Encephalitis is characterized by inflammation of the brain resulting in fever, seizures, and alterations in EEG patterns, and has been known to occur as result of either auto-immune diseases or viral infections [205, 206], including SARS-CoV-2 [207]. Furthermore, there have been cases of seizures and ischemic stroke in infected patients ([208–211]). Stroke is an acute focal lesion in the CNS that leads to loss of neurological function. It has a vascular origin and is characterized by abrupt interruption of blood supply in parts of the brain [212]. These very common events in COVID-19 patients are caused by coagulopathy and cerebral endothelial cells damage. Furthermore, disruption of ACE2 function caused by high SARS-CoV-2 viremia can impair its vasoprotective function in brain vessels [77]. These symptoms appear to be more commonly found in the elderly, strongly correlating aging with more severe outcomes of SARS-CoV-2 infection [70].

Several studies demonstrated a direct correlation of CNS invasion of SARS-CoV-2 and neurological impairment [202, 213]. In contrast, other reports were unable to detect viral RNA in the CSF of COVID-19 patients experiencing neurological complications [214, 215], suggesting that COVID-19 related neuropathies may not occur due to a viral invasion of the CNS. Overall, it appears that SARS-CoV-2 could be acting as an immune trigger, and that encephalitis caused by the virus may occur due to an exacerbated immune reaction, rather than direct viral penetration of the tissue. However, it is important to clarify that both pathways are possible, and the ending result is likely some combination of both factors.

As stated previously, productive endothelial infection by SARS-CoV-2 appears to be limited and was not confirmed in in vitro studies on human endothelial cells [83]. On the other hand, activation of coagulation pathways during viral infection may lead to excessive production of pro-inflammatory cytokines, leading to altered function of the BBB as well as thrombosis that can cause direct damage to blood vessels [216]. For instance, IL-6 is a known modulator of BBB function, being able to modulate nitric oxide signaling as well as promoting angiogenesis trough the HIF1-α/VEGF pathway (Andreozzi et al. [217], Fu et al. [218]). Furthermore, COVID-19 patients with neurological symptoms show altered inflammatory mediators such as IL-6 and TGF-β in both serum and CSF when compared to patients with only mild symptoms [219, 220]. Other groups observed elevated levels of IL-1, IL-8 INF-γ and TNF-α in SARS-CoV-2-infected individuals ([203], Farhadian et al. [221]; Velpula et al. [222]). Increased levels of inflammatory cytokines correlate with disease severity ([43], Del Valle et al. 2020; Han et al. 2020) and the presence of neurological symptoms [220]. Interestingly, a recent study demonstrated that SARS-CoV-2 can infect epithelial cells more efficiently in vitro if pre-treated with IFN-γ [125].

The described sepsis-like cytokine storm can be overly harmful to the host if not properly mitigated [223–225]. In fact, similar cytokine storm events were observed in previous coronavirus-triggered diseases [226, 227]. The exact source of cytokines induced by SARS-CoV-2 is not yet clear and is likely to be derived from multiple cellular sources. While productive infection of endothelial cells may be limited, it is possible S1 protein alone could have detrimental effects on BBB integrity, which would also facilitate infection of the CNS [26]. Lastly, the virus may invade the CNS by infecting other brain cells that express high levels of ACE2.

An alternative mechanism by which SARS-CoV-2 may penetrate the BBB is not by direct infection of surrounding cells and resulting inflammation, but rather activation of signaling pathways trough viral proteins. Indeed, S1 protein can significantly alter the expression of proteins such as ZO-1 and Claudin-5, which are integral members of the endothelia tight-junctions that, when altered, directly impact endothelium permeability [24, 26]. In COVID-19 patients, cellular components released into the blood (e.g., viral proteins, RNA, and debris in general) as a result of a cytolytic event could be harmful not only by directly increasing the risk of coagulopathy and stroke [228] but also by indirectly increasing BBB permeability, allowing the virus to infect astrocytes, which do express high levels of ACE2 and TMPRSS2 [26].

## Current and/or promising therapies to target aged BBB in COVID-19

Several data indicate that the highest prevalence of severe cases or deaths caused by SARS-CoV-2 infection occurs in elderly patients, which may be due to a higher incidence of comorbidities with associated vascular disorders, such as hypertension, diabetes and cardiovascular diseases [229]. Indeed, aging is accompanied by cerebrovascular dysfunctions, which can increase the risks for ischemic stroke, intracerebral hemorrhages and cognitive decline [230]. As previously mentioned, the cytokine storm observed in COVID-19 results in increased production of pro-inflammatory cytokines, and has been associated with high morbidity in patients. The increase in these pro-inflammatory mediators is also observed in the blood plasma of healthy elderly individuals [231], suggesting that inflammaging may be relevant to increased vulnerability of elderly patients to viral infection [232]. Inflammaging [118] is a characteristic phenotype of normal brain aging that has also been closely linked to many age-related diseases [14, 231]. Anti-inflammatory therapies have been proposed to mitigate the COVID-19-associated cytokine storm syndrome. Among such proposed approaches, the use of Tocilizumab was recently recommended in hospitalized patients who present respiratory decompensation due to SARS-CoV-2 infection [233]. Tocilizumab is a humanized monoclonal antibody, clinically used in rheumatological disorders, that inhibits IL-6 signaling through the competitive blocking of its receptor binding site (IL-6R) [234]. IL-6 is a pro-inflammatory cytokine produced by different brain cells that is upregulated in neuroinflammatory conditions, such as infections and CNS injuries [235]. Recent evidence suggests that the use of Tocilizumab combined with dexamethasone, an anti-inflammatory agent, promotes modest benefits in reducing mortality of hospitalized patients with severe COVID-19 who have increasing oxygen needs [236–238]. Elevated levels of IL-6 promote endothelial dysfunction and increased vascular permeability through the modulation of junction proteins, such as VE-cadherin and ZO-1 [239, 240]. Thus, the use of anti-IL-6 recombinant monoclonal antibodies has the potential to attenuate inflammation-induced endothelial activation and provide a therapeutic strategy to reverse cerebrovascular dysfunctions associated with COVID-19 infection. However, there is still insufficient data to support the benefits of this therapy for this group of patients.

Since BMEC dysfunctions could also be caused by direct infection and replication of SARS-CoV-2 in the CNS, antiviral therapies emerge as therapeutic strategies to mitigate neurological manifestations in patients with COVID-19. Remdesivir is an antiviral drug that suppresses the rapid replication of SARS-CoV-2 [241] and has shown benefits in reducing recovery time in adults hospitalized with COVID-19 [242]. Antiviral therapy with remdesivir combined with dexamethasone has been recommended as another strategy to mitigate infection and the inflammatory response in hospitalized COVID-19 patients who require increasing amounts of oxygen [243].

As stated above, COVID-19 is accompanied by dysfunctions in the vascular endothelium and induction of a procoagulant state with elevation of thrombotic markers, such as d-dimers. Elevated levels of d-dimer can trigger ischemic stroke and have been associated with worse clinical outcomes in patients with COVID-19 [244]. Several studies have explored the action of anticoagulants, such as low molecular weight heparin, as a therapeutic strategy to reduce the risk of thrombotic disease in patients with COVID-19. A retrospective study showed that low molecular weight heparin appears to be associated with a better prognosis in hospitalized COVID-19 patients with coagulopathy or with high d-dimer [245]. In addition to its anticoagulant properties, heparin is known to have anti-inflammatory effects on endothelial cells, leading to reduced translocation of nuclear factor kappa B (NF-κB) transcription factor and production of inflammatory markers, such as IL-1β, IL-6, E-selectin, and intercellular adhesion molecule (ICAM)-1 [246], which could positively impact the inflammation associated with COVID-19. Unfortunately, there is still insufficient evidence on the effectiveness of the use of anticoagulant therapies in the treatment of cerebral vascular disorders due to COVID-19.

A strategy recently approved by the Food and Drug Administration (FDA) that has the potential to mitigate the risks of severe COVID-19 progression in elderly patients or those with associated vascular comorbidities was the combination of bamlanivimab and etesevimab. Bamlanivimab and etesevimab are neutralizing human monoclonal antibodies, which specifically bind to different epitopes in the SARS-CoV-2 Spike protein receptor-binding domain (RBD), blocking the binding of the virus to the ACE2 receptor on the host cell surface. These antibodies have been approved for use in adult and pediatric outpatients who are at high risk of progressing to severe COVID-19 and/or hospitalization. Individuals who are most at risk, according to the criteria adopted by the FDA, include elderly patients aged ≥ 65 years or ≥ 55 years who have comorbidities, such as cardiovascular disease and hypertension [247]. As aging progresses, the risk of being hospitalized as a result of COVID-19 increases. Combined administration of bamlanivimab and etesevimab appears to significantly reduce the risk of hospitalization and death related to COVID-19 [248].

Overall, the rapid expansion of knowledge of SARS-CoV-2 virology has stimulated the development of a number of clinical trials adopting anti-inflammatory, anti-thrombotic, and antiviral approaches. The effectiveness of these approaches must be assessed individually, due to the different conditions associated with patients’ susceptibility, such as age and the presence of pre-existing endothelial dysfunctions. Thus, further studies are needed to precisely determine the benefits of current therapies for brain endothelial dysfunction in elderly patients.

## Concluding remarks

COVID-19 hit the world as an unprecedented pandemic, followed by an impressive mobilization of the scientific community in order to rapidly understand the mechanism involved in physiopathogenesis of this viral disease. The severity of acute disease urged for studies about invasiveness and abnormalities in different organs, due to the wide range of clinical manifestations, which include lethal pneumonia and respiratory failure, and can promote important neurological dysfunctions that, in many cases, are perennial. While respiratory deficit dominates the clinical symptoms of the acute form of the disease, the cerebrovascular impact of COVID-19 plays an important role in the pathology of the infection due to the loss of vascular stability, which is directly related to endothelial activation by the cytokine storm, being aging a risk factor for COVID-19 progression. One of such challenges is the understanding of the post-COVID syndrome and the onset of neurological manifestations.

**Fig. 1:**
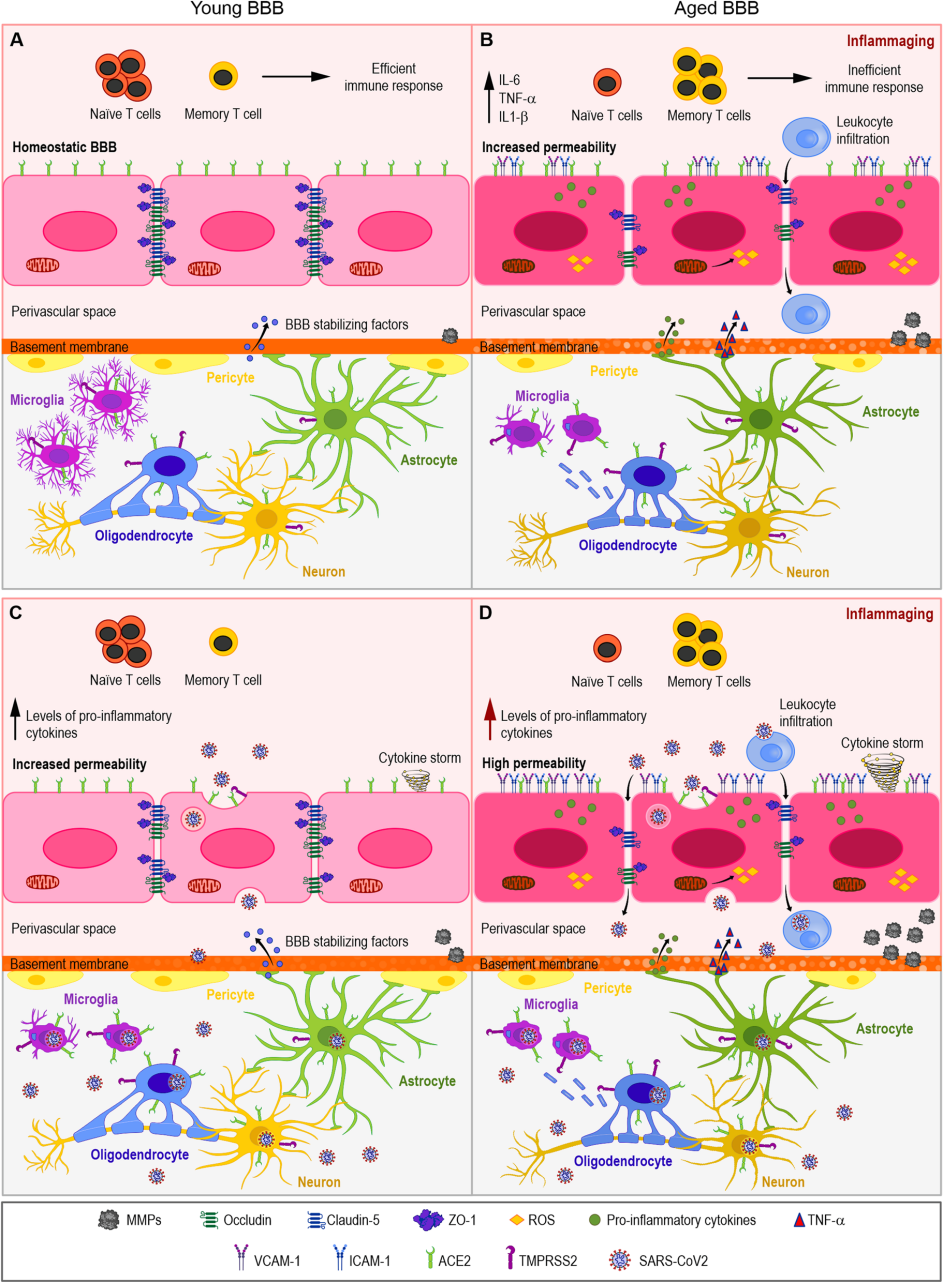
Proposed model of aging as a cofactor to COVID-19 neuropathogenesis. Neural cells express variable levels of SARS-CoV-2 receptors, such as ACE2 and TMPRSS2, shown in BMECs, microglia, astrocytes, neurons and pericytes (**A**). Young brain parenchyma is protected by the BBB, with low paracellular transport and high levels of TJ proteins, including zona occludens, claudins and occludins (**A**). Aged BBB (**B**) displays increased permeability as well as increased expression of adhesion molecules such as ICAM-1 and VCAM-1 and the phenomenon of inflammaging, that includes increased levels of pro-inflammatory cytokines (IL-6, TNF-α and IL-1β), reduction of circulating Naïve T cells and increase in memory T cells and mitochondrial dysfunction (**B**). SARS-CoV-2 infection of young hosts induce cytokine storm (**C**), destabilization of the BBB and increase in circulating and local levels of pro-inflammatory molecules, basement membrane abnormalities due to increased MMPs and infection of neural cell types, including neurons, oligodendrocytes, microglia and BMECs. Aged BBB (**D**) may display highly increased permeability and endothelial activation, with expression of adhesion molecules, striking presence of cytokine storm with neuronal loss or atrophy, demyelination and increased microglial activation

Aging leads to a significant shift in immunological response, hence increasing susceptibility to COVID-19 and decreased response to immunization. Among hallmarks of inflammaging, we highlighted in this review the changes to mitochondria biology and plasticity, that further contribute to a constant inflammatory state (Fig. 1A, B). Brain endothelium loses its barrier properties, leading to “leaky” BBB, which can also contribute to higher susceptibility to SARS-CoV-2 neuroinvasion. In the brain parenchyma, neural cells express higher levels of SARS-CoV-2 receptors (Fig. 1B), such as ACE2 and TMPRSS2.

Such pre-inflamed scenario can lead to an aggravation of the cytokine storm phenomenon (Fig. 1D), which may lead to more severe overall pathology in COVID-19. Even though hundreds of studies have been published, in the past 2 years, describing factors related to the prevalence of COVID-19 and risk factors, “age” is certainly one of the most relevant factors and still is the focus of several studies in progress.

COVID-19 still poses a challenge even after vaccination campaigns have reached high levels in many countries. However, the different variants of concern of SARS-CoV-2 that have emerged since then, and reinfection cases are very common. In this context, further studies investigating the mechanisms of invasion and induction of BBB dysfunctions are necessary for the development of therapeutic strategies to treat neurological sequelae post-infection.

## Data Availability

Not applicable.
